# Translation, validity and reliability of the persian version of the rapid assessment of physical activity questionnaire

**DOI:** 10.1186/s12877-024-05065-3

**Published:** 2024-05-23

**Authors:** Majid Barati, Eva Ekvall Hansson, Zahra Taheri-Kharameh, Tari D Topolski

**Affiliations:** 1 Autism Spectrum Disorders Research Center, Hamadan University of Medical Sciences, Hamadan, 6517838695 Iran; 2 Department of Public Health, Asadabad School of Medical Sciences, Asadabad, Iran; 3 Department of Health Sciences, Lund University, Lund, Sweden; 4 Spiritual Health Research Center, School of religion and health, Qom University of Medical Sciences, Qom, Iran; 5 Department of Public Health, School of Health, Qom University of Medical Sciences, Qom, Iran; 6 Department of Health Services, University of Washington, Seattle, WC 89101 Iran

**Keywords:** Translation, Validity, Reliability, Motor activity, Geriatric, Scale

## Abstract

**Purpose:**

The purpose of this study was to produce a valid and reliable Persian version of the Rapid Assessment of Physical Activity (RAPA) questionnaire, which previously has been shown to be valid and reliable for assessing physical activity among older adults.

**Methods:**

Permission was obtained from the scale developer, who provided a copy of the the Linguistic Validation of the RAPA Qestionnaire, which utilizes a forward-backward translation methodology. Content validity, face validity, and construct validity of the questionnaire were then determined. Comparison of known groups (older adults with more or less than 50% balance confidence) was used to assess construct validity and the Leiden-Padua (LEIPAD) quality of life questionnaire were used to assess convergent validity. Three hundred older adults, who were members of the Qom retirement centers, participated in the study. Thirty participants completed the RAPA twice with a one-week interval to determine test–retest reliability.

**Results:**

Results of comparisons of known groups showed that the mean RAPA score of the older people with greater balance confidence was significantly higher. Significant correlations between most of the scores obtained from both RAPA and the LEIPAD questionnaires confirmed the convergent validity of the questionnaire. Intraclass Correlation Coefficient (ICC) was as high as 0.94 showing that the test–retest reliability was good.

**Conclusion:**

This study showed the Persian RAPA is a reliable and valid instrument for measuring physical activity among older individuals in both research and clinical contexts.

## Introduction

Effects of age, the progressive declines in physiological function, are associated with mobility impairments and increased dependence. Regular physical activity (PA) can bring significant health benefits to people of all ages, especially those who are aging. Research has increasingly showed that PA can increase active and independent years of life, reduce risk of chronic condition and disability, and improve quality of life [1, 2]. The World Health Organization (WHO) defines physical activity as any physical activity produced by the musculoskeletal system that requires energy consumption, and estimates physical inactivity is an independent risk factor for chronic diseases and the cause of 1.9 million deaths worldwide. The recommendations from WHO include both aerobic exercise and strength exercise as well as balance exercises to reduce the risk of falls [3].

Understanding the amount and type of physical activity of individuals is essential for health promotion planning [4]. Questionnaires are one of the data collection methods commonly used to evaluate PA in research as well as in clinical practice [5]. Compared to alternative methods of evaluating PA, questionnaires are short, easy and with minimal financial resources as a screening tool. Standard physical activity assessment tools for the Iranian older people`s use are limited and relatively long [6].

The Rapid Assessment of Physical Activity (RAPA) is a self-administered questionnaire designed by Topolski et al., in 2006 to examine levels of physical activity. This questionnaire is short and easy to understand and has been widely used in many studies. Another advantage of RAPA is the examination of the strength and flexibility activities that are important to reduce the risk of falls in the older people [7].

Over the past decade, the RAPA has been utilized in numerous studies as a valid measure of PA [12]. The translation and validation of this tool also have been extensively studied by researchers in various countries, such as Portugal [8], Turkey [9], Spain [10] and Austria [11]. These studies have shown that the RAPA can be considered a reliable and valid tool for assessing physical activity. Despite its translation and validation in multiple languages, it is worth noting that a validated version of the RAPA in Persian has not yet been developed in Iran, to the best of our knowledge. The aim of this study was to determine the validity and reliability of the Persian version of RAPA in the older people.

## Method

### Design and participants

This is a methodological study to determine the psychometric characteristics of the RAPA, as a quickly assessing the level of physical activity in older people in Qom, a provincial city in the central region of Iran. In psychometric studies, it is recommended to have a sample size of at least 300 individuals [13]. So, a total of 300 older individuals were selected to participate in this study based on specific criteria. The inclusion criteria included people aged 60 years or older, members of Qom’s retirement centers, independently living at home, lack of psychological and cognitive impairment, consent to participate in the study, ability to communicate and respond. The exclusion criterion was as follows: refusal for participation in study. The purpose of the study was explained to the participants.

### Translation procedures

After obtaining permission from the scale developer through correspondence, we proceeded to utilize the recommended Forward-Backward method for the translation process. The translation was conducted based on the guidelines provided by the WHO, as part of the International Quality of Life Assessment Project (IQoLAP) [14]. This approach to translation and validation has been developed for use with the SF-36, but it can also be applied to other translation efforts. The translation process involved two translators: one without medical knowledge and another who was a medical university expert. By comparing the results of both translations, we synthesized a final version, which was then translated back into English by an independent translator. This translated copy was then sent to the original RAPA developer for review. After incorporating necessary modifications based on the developer’s feedback, the final Persian version was approved.

### Content validity

In order to assess the content validity of the questionnaire, 10 experts specializing in geriatrics, physical activity, and psychometrics were invited to participate. They were asked to complete the questionnaire and provide feedback based on the content validity index (CVI) and the content validity ratio (CVR). To evaluate the CVR, three items were considered for each question that were: (a) it is necessary; (b) useful but not essential; and (c) not necessary. Panel members selected one of the three options for each item of the scale. Then, the CVR of the RAPA was calculated as follows CVR= (Ne - N/2)/(N/2), in which the Ne is the number of experts evaluating “essential” and N is the total number of experts. The determination of the accepted value was made by referring to Lawshe’s table and considering the number of experts involved. As per Lawshe’s table, an item with a CVR exceeding 0.62 is considered acceptable when there are 10 experts involved [15].

Then, to assessment the CVI, 3 criteria, simplicity, specificity, and clarity were individually examined in a 4-part Likert scale for each item by 10 experts (e.g. for simplicity = 1, incomprehensible = 4, Quite simple and understandable). For this purpose, in this study, the CVI score will be calculated by summing the percentages of agreement scores for each item that scored 3 and 4 (highest score) on the total number of experts. To determine the suitability of items, we relied on the CVI. Items with a CVI above 0.79 were found to be highly suitable, indicating a strong alignment with the desired criteria. For items falling within the range of 0.70 to 0.79, modifications were deemed necessary to enhance their suitability. Lastly, items with a CVI below 0.70 were deemed unacceptable, as they did not meet the desired criteria [16].

### Face validity

In order to determine face validity, 10 older people with inclusion criteria into research were asked to express for content, clarity, readability, and simplicity and easy to understand tool expressions. Finally, according to patients` feedback and research team, necessary changes were considered; the target population of this study was the older people members of retirement centers in Qom. Sampling method was stratified and inclusion criteria were age equal to and over 60 years, home living, lack of mental and cognitive impairment (score of 6 or above in Farsi version of abbreviated mental test), consent to participate, capable of communicating and responding.

After obtaining permission from the Qom University of Medical Sciences (IR.MUQ.REC.1400.135) and coordinating with retirement centers, Questionnaires were completed within 6 months after the confidentiality of the information was obtained and the participants` consent was obtained.

### Instruments

Demographic and medical information, RAPA, Leiden-Padua (LEIPAD) questionnaire and Activities-Specific Balance Confidence scale (ABC) were used to collect data.

RAPA is a self-report physical activity measurement tool originally designed in English in the USA. It contains two sections and 9 items with response yes/no options. The first part of the tool contains 7 items measuring different levels of physical activity. The second part questions the strength and flexibility training. To score the RAPA, the question with the highest score with a “yes” response is chosen from the first items. In addition, an affirmative answer for participating in muscle strengthening activities such as lifting weights or calisthenics gets one point. The older people, who participated in flexibility activities such as stretching or yoga, were awarded two bonus points, leading to a total possible score of 3 points. The reliability and validity of the original version are confirmed [7].

Quality of life was assessed by using LEIPAD, an Internationally Applicable Instrument to Assess Quality of Life in the older people. This questionnaire was designed by the WHO sponsored by Di Leo et al., and measures the quality of life of the older people in 7 dimensions of physical function (5 questions), self-care (6 questions), depression and anxiety (4 questions), cognitive functioning (5 questions), social function (3 questions), sexual function (2 questions) and life satisfaction (6 questions). It is designed as a Likert and each question has four options ranging from zero (worst case) to three (best case) and has a total of 31 questions with a minimum of 0 and a maximum of 93 [17]. The validity and reliability of the questionnaire have been confirmed by Maliki et al. [18].

Balance confidence was measured using the short form of the ABC [19]. This scale requires participants to rate their confidence in their balance on a scale from 0% (no confidence) to 100% (completely confident) for six different activities of daily living. The overall balance confidence score was calculated as a percentage based on the average of all six items on the ABC-6. Higher scores on the scale indicate greater confidence in one’s balance. A score below 50% on the ABC-6 suggests lower levels of functioning and confidence in maintaining balance. The short version of the scale, known as ABC-6, is a more simplified and concise version compared to the original ABCscale [20], as suggested by previous studies [21, 22]. The Persian version of the ABC-6 scale has been validated and shown to be reliable in measuring balance confidence [23].

Demographic and disease information questionnaire include age, gender, marital status, residence status, education level, and economic status.

### Construct validity

#### Known group comparison

Known Group Comparison was used to determine construct validity in this study. This type of validity determines the ability of a tool to differentiate respondents according to a criterion and assumption. In this study, the parameter used was balance confidence in the older people. For this purpose, comparison of RAPA score between two groups of older people with balance confidence was more or less than 50%. We expected people with higher levels of confidence in balance to score better on the RAPA than older people with no confidence in balance [24]. The Cohen’s d statistic was utilized to compare two groups and assess the magnitude of the effect size.

#### Convergent validity

In order to investigate convergent validity, the correlation between the RAPA scores and LEIPAD scores were measured. We hypothesized that there would be a positive significant correlation between the RAPA and the LEIPAD. In assessing the strength of the correlation, we utilized a Pearson’s correlation.

### Reliability

One weeks after the initial survey, the RAPA was once again distributed to 30 participants who had previously responded to the first set of questionnaires. These participants had willingly agreed to complete the RAPA twice, with a one-week gap between administrations. The random method was employed to select the participants for this procedure. The purpose of this procedure was to assess the reliability of the questionnaire using the test-retest method [25].

### Data analysis

Data analysis was performed using SPSS16 software at the significant level of 0.05. Sample characteristics and the RAPA score were analysed by using descriptive statistics. As known group comparison, the RAPA score of participants with balance confidence more or less than 50% were evaluated with the independent t-test. To assess the concurrent validity of the RAPA, Pearson’s correlation coefficient between the scores of the RAPA and LEIPAD was computed. A coefficient ranging from 0 to 0.29 indicates a weak correlation. A coefficient between 0.30 and 0.69 suggests a moderate correlation. Finally, a coefficient between 0.70 and 1 indicates a strong correlation [26]. Test-retest reliability was assessed by computing the intraclass correlation coefficient of each domain. If the index is above 0.80, the stability level is favorable [25].

## Results

### Sample characteristics

Mean and standard deviation of the participants’ age was 64.6 ± 5.24 years. Most participants were males (77.7%), married (88.7%), and had low literacy (58.6%). Just 26% of the participants were regularly active according to the RAPA. Most of the participants (71.7%) reported that they did not do strength and/or flexibility training. The demographic characteristics of participants are presented in Table 1.

**Table 1:** Socio-demographic information of the samples (*N* = 300)

	*N*	(%)
**Age** (years)		
Mean (SD)	59.46	11.24
**60–64**	180	60.0
**65–69**	69	23.0
**≥ 70**	51	17.0
**Gender**		
Males	233	77.7
Females	67	22.3
**Educational status**		
Illiterate	77	25.3
Primary school	100	33.3
Secondary school	21	7.4
High school	39	13.0
University	63	21.1
**Marital status**		
Married	266	88.7
Divorced/ widowed	34	11.3
**Economic status**		
Poor	211	70.3
Good	89	29.7
**Smoking status**		
Smoker	71	23.7
Non-smoker	229	76.3
**Body mass index (kg/m** ^**2**^ **)**		
Under weight (< 18.5)	36	12.0
Normal weight (18.5–24.9)	188	62.6
Overweight (≥ 25.0)	59	19.6
**Comorbidity** (yes)	225	75.0

### Content and face validity

Content validity was assessed for CVI and CVR, and all items achieved satisfactory scores. The overall tool demonstrated a CVI value of 0.96 and a CVR value of 0.94. Furthermore, individual item CVR scores surpassed 0.60, while item CVI scores were above 0.8. The participants approved all 10 items in terms of face validity.

### Construct validity

With regard to the known group comparison, shown in Table 2, the older people with higher confidence exhibited significantly higher RAPA scores than those who had poor confidence.

**Table 2:** Known-group comparison of the RAPA

Domain score	ABC < 50 M(SD)	ABC > 50 M(SD)	95% Confidence interval of the difference	Effect size Cohen’s d	*P*-value
Aerobic	3.34 (2.06)	4.36 (2.07)	-1.49, -0.46	0.93	0.0001
Strength & flexibility	0.51 (1.04)	0.72 (1.12)	-0.50, 0.05	0.43	0.051
RAPA	3.82 (2.81)	5.09 (2.78)	-1.94, -0.52	0.89	0.0001

### Convergent validity

The correlation between RAPA and LEIPAD is shows in Table 3, which was used to assess convergent validity. The correlation coefficient of the two questionnaires was positive and significantly in all dimensions except for social function and sexual function.

**Table 3:** Correlation between the RAPA and Liepad

Variable	Physical functioning	Self-care	Depression and anxiety	Cognitive functioning	Social functioning	Sexual functioning	Life satisfaction	Total
Aerobic	0.373^**^	0.199^**^	0.189^**^	0.181^**^	0.098	0.066	0.161^**^	0.300^**^
Strength & flexibility	0.261^**^	0.132^*^	0.180^**^	0.107^*^	0.084	0.086	0.131^*^	0.234^**^
RAPA	0.379^**^	0.199^*^	0.213^**^	0.167^**^	0.106	0.095	0.176^**^	0.313^**^

** Correlation is significant at the 0.01 level

* Correlation is significant at the 0.05 level

### Reliability

To assess test-retest reliability, the ICC for the RAPA was calculated 0.94 and interpreted as having very good test-retest reliability. The ICC also exceed 0.95 and 0.91 for aerobic part and strength and flexibility part, respectively (Table 4).

**Table 4:** Reliability of the RAPA-P

Domain score	Item number	ICC	*P*-value
RAPA 1 (Aerobic)	7	0.95	0.001
RAPA 2 (strength & flexibility)	2	0.91	0.001
RAPA	9	0.94	0.001

## Discussion

The aim of this study was to assess the psychometric properties of the RAPA in Iranian older people. Many studies have been conducted to assess and increase PA in older people. However, an absolute prerequisite of these studies is the availability of a short, valid and reliable instrument. In the current study, the questionnaire was translated by experienced and skilled experts who followed the principles of translation and ensured accuracy in cultural adaptation. The study strictly adhered to the recommended steps for instrument translation and cultural adaptation of the translated version. Content validity was assessed using CVI and CVR, and all items received satisfactory scores. Face validity was qualitatively confirmed through feedback from 10 older adults.

In this study, to evaluate the construct validity of the questionnaire, we used the method of known groups comparison for balance confidence parameter. The results showed that RAPA score was significantly lower in older people with confidence less than 50% as expected. This assumption in the study was confirmed. Another study that showed balance confidence is a main determinant of physical activity levels in the older people with diabetes [27]. These results are consistent with those of the Spanish version of RAPA, which also showed that physical activity was significantly and inversely correlated with BMI and waist circumference [28].

The correlation between the scores obtained from the RAPA and LEIPAD questionnaires varied from low to moderate, suggesting a positive correlation of the same magnitude. This finding supports the concurrent validity of the questionnaire, aligning with the results of previous studies conducted in this field. CHAMPS was used to assess the validity of the original version of RAPA and the results showed there is a significant correlation between RAPA and CHAMPS (*r* = 0.54). Results of the Portuguese version of RAPA showed that lower levels of physical activity were associated with worse self-report disability and slower speed [8]. Validity of the Mexican-American-Spanish version of RAPA was determined by assessing the correlation between RAPA data and the accelerometer as a direct measure of physical activity level. There was a significant relationship between RAPA and moderate and vigorous minutes of physical activity, indicating RAPA validity [10]. The Turkish version of the RAPA showed acceptable concurrent validity, because there were positive correlations between the RAPA, International Physical Activity Questionnaire- Short Form and Physical Activity Scale for the older people [9]. Although we did not use the direct physical activity tools mentioned in other studies, the present results also showed that RAPA was significantly associated with health outcomes.

In this study, the test-retest reliability of the RAPA was assessed. ICC for the RAPA was calculated to be 0.94, indicating very good test-retest reliability. In the original study of the RAPA, the test–retest was not evaluated. But in Turkish version, the weighted kappa coefficients exceeded 0.81 for both parts of RAPA, the aerobic score and strength and flexibility score, showing that the test–retest reliability was very good [9]. In contrast, the Chilian version, was not an authorized translation, and did not follow the prescribed translation and validation process required by the developer, exhibited an ICC that was lower than the favorable stability. As did the Portuguese version, which showed a moderate test–retest reliability with a weighted κ = 0.67 [8].

Based on the findings of this study, RAPA is a reliable and valid instrument that features a short completion time, capability of use in different settings, simple scoring, and suitable reliability and validity, and is a useful tool.

This study had limitations. First, all of the tools used to assess the validity were self-report.

Although these questionnaires were recognized as valid and standard tools, objective tools such as accelerometers or pedometers may provide more accurate information on the validity of the Persian version of RAPA. Second, sampling carried out only in the retirement centers in this study reduces the generalizability of the findings. In future studies, it is also recommended that researchers focus on the sensitivity and specificity of the questionnaire.

## Conclusions

The findings of this study suggest that the RAPA questionnaire has good psychometric properties for use with older Iranian adults. The RAPA was originally developed for use by genentologist to prompt talking with their patients about the need to engage in physical activity, however, it has been shown to be is appropriate for use to measure amount and type of physical activity as well as health outcomes in both research and clinical settings.

## Data Availability

The datasets utilized and/or analyzed during the present study are available from the corresponding author upon reasonable request.

## Abbreviations

ICC: Iterative reliability coefficient
RAPA: Rapid Assessment of Physical Activity
ABC: Activities-Specific Balance Confidence scale
LEIPAD: Leiden-Padua
